# Inorganic arsenic in seaweed: a fast HPLC-ICP-MS method without coelution of arsenosugars

**DOI:** 10.1007/s00216-024-05250-8

**Published:** 2024-03-23

**Authors:** Rebecca Sim, Marta Weyer, Ásta H. Pétursdóttir

**Affiliations:** 1 Public Health and Food Safety, Matís, Vínlandsleið 12, 113 Reykjavík, Iceland; 2 Faculty of Physical Sciences, University of Iceland, Dunhagi 3, 107, Reykjavík, Iceland; 3 Department of Chemistry, University of Aberdeen, Meston Walk, Aberdeen, Scotland

**Keywords:** Inorganic arsenic, Seaweed, Arsenic, Arsenosugars, HPLC-ICP-MS, DOE

## Abstract

Seaweed is becoming increasingly popular in the Western diet as consumers opt for more sustainable food sources. However, seaweed is known to accumulate high levels of arsenic—which may be in the form of carcinogenic inorganic arsenic (iAs). Here we propose a fast method for the routine measurement of iAs in seaweed using HPLC-ICP-MS without coelution of arsenosugars that may complicate quantification. The developed method was optimised using design of experiments (DOE) and tested on a range of reference materials including TORT-3 (0.36 ± 0.03 mg kg^−1^), DORM-5 (0.02 ± 0.003 mg kg^−1^), and DOLT-5 (0.07 ± 0.007 mg kg^−1^). The use of nitric acid in the extraction solution allowed for the successful removal of interferences from arsenosugars by causing degradation to an unretained arsenosugar species, and a recovery of 99 ± 9% was obtained for iAs in Hijiki 7405-b when compared with the certified value. The method was found to be suitable for high-throughput analysis of iAs in a range of food and feed matrices including *Asparagopsis taxiformis* seaweed, grass silage, and insect proteins, and offers a cost-effective, fast, and robust option for routine analysis that requires minimal sample preparation. The method may be limited with regards to the quantification of dimethylarsenate (DMA) in seaweed, as the acidic extraction may lead to overestimation of this analyte by causing degradation of lipid species that are typically more abundant in seaweed than other marine matrices (i.e. arsenophospholipids). However, the concentrations of DMA quantified using this method may provide a better estimation with regard to exposure after ingestion and subsequent digestion of seaweed.

**Supplementary Information:**

The online version contains supplementary material available at 10.1007/s00216-024-05250-8.

## Introduction

Arsenic is a naturally occurring element released from the erosion of mineral-bearing rocks and anthropogenic activities such as smelting and mining. The toxicity of arsenic-containing compounds is highly dependent on chemical speciation and oxidation state, with the inorganic species being classified as carcinogenic by the International Agency for Research on Cancer (IARC) [1]. The sum of the inorganic species, arsenite and arsenate, is often referred to as inorganic arsenic (iAs) and exposure has been linked to bladder, lung, and skin cancers, as well as diabetes, cardiovascular disease, and pulmonary disease [2–9]. Ingestion of food or drinking water is the major exposure route to iAs, with rice and cereals being significant contributors to dietary intake [10]. Seafood may also contribute to dietary intake depending on cultural and geographic differences in consumption [10, 11]. The speciation analysis of seafood matrices can be particularly challenging due to the number of components that require separation from iAs, such as arsenobetaine (AB), arsenosugars (AsSugars), and small, methylated arsenicals like dimethylarsenate (DMA) and monomethylarsenate (MMA). Seaweeds are widely known to contain high levels of arsenic—predominantly in the form of AsSugars where they may comprise over 85% of the water-soluble As [12]. High concentrations of these sugars can hinder the quantification of iAs species by coelution during chromatographic analyses (i.e. anion exchange). Sample introduction techniques such as hydride generation (HG) have been developed to overcome this issue but involve the use of strong oxidising agents such as NaBH_4_ under acidic conditions which may be hazardous—as well as additional apparatus [13]. Alternatively, separation of these closely eluting analytes using anion exchange chromatography often requires long runtimes or the use of a mobile phase gradient [14, 15]. However, previous studies have shown that using a low concentration of a protic acid in the extraction step can cause hydrolysis of the R-group of the ribose ring, producing an AsSugar that elutes in the void volume of the anion exchange column, thus simplifying the chromatography [16]. The use of nitric acid in the extraction solution has been successfully applied to seafood and rice matrices, and has also been shown to increase the recovery of iAs from seaweed [17, 18].

Additionally, design of experiment (DOE) can be used to optimise chromatographic separation and runtime, whereby a systematic approach is taken to model the effects of a large number of factors in fewer runs. DOE can be used for method development, optimisation, or robustness testing, and has previously been applied to optimise the extraction of arsenic species in seafoods, rice, and soils [15, 19, 20], however, is most commonly used with pharmaceutical analyses [21]. Optimisation of extraction parameters such as time, volume of solvent, and temperature has been previously reported, as well as instrumental variables such as flow rate, column temperature, and pH [15, 22].

Whilst in 2015 the EU introduced regulations for maximum levels of iAs allowed in rice and derived products, similar legislation is yet to be proposed for seaweed for human consumption. France is currently the only European country to have implemented nationwide maximum levels for iAs, currently set at 3 mg kg^−1^ for seaweed condiments, and in Australia and New Zealand, this limit is even lower at 1 mg kg^−1^. Additionally, an updated risk assessment by the European Food Safety Authority (EFSA) in 2024 determined that current exposure levels to inorganic arsenic raised health concerns for adult consumers—highlighting the need for the investigation of iAs levels in foods for human consumption [23]. Seaweed poses a unique challenge due to the large variations in iAs concentration between species. For example, in *Laminaria spp*., the total As content can reach upwards of 100 mg kg^−1^, and in *Laminaria digitata* specifically iAs can comprise over 50% of this total; however, in *Laminaria longicruris*, iAs has been shown to account for less than 1% of the total As [18, 24, 25]. As such, the implementation of maximum total As limits would not be nearly specific enough to assess the full toxicological risk posed by the consumption of seaweed.

There is an increasing demand for sustainable, alternative food sources in an effort to reduce the effects of climate change, and as such seaweed production is now the fastest-growing aquaculture sector [26]. Besides being a popular health food with a high nutrient content, seaweed is also finding increased uses in the food production, cosmetics, and pharmaceutical industries [27–29]. Although the current iAs levels in the majority of seaweed species pose no imminent threat to human health, outliers such *Laminaria digitata* or *Sargassum fusiforme* (hijiki) mean it is crucial that levels are monitored, and occurrence data is gathered as the consumption of seaweed and derived products becomes more widespread [30]. Here, we propose a fast chromatographic method for the routine measurement of iAs in seaweed suitable for high-throughput analysis and use across a wide range of food and feed matrices. DOE was used to optimise the acid concentration and chromatographic parameters to achieve an acceptable separation of As(V) from other analytes in under 7 min.

## Experimental

### Chemicals and standards

All chemicals used were of analytical grade or better and Milli-Q water from a Millipore water dispensing system (Millipore, France) was used throughout unless otherwise stated. Hydrogen peroxide (H_2_O_2_) (for trace analysis, ≥30%) and nitric acid (HNO_3_) (ROTIPURAN Supra, 69%) were supplied by Supelco (Sigma-Aldrich, France) and Carl Roth (Germany) respectively. Ammonium carbonate was obtained from BDH (UK) and methanol (HPLC grade) was purchased from Honeywell (USA). Arsenate standards used for external calibration of the iAs analysis were prepared from a stock solution of disodium hydrogen arsenate heptahydrate (98.5%) supplied by Argus Chemicals (Italy) dissolved in Milli-Q water. DMA and MMA standards used for spiking experiments were prepared from dimethylarsinic acid disodium salt (100%) supplied by Argus Chemicals (Italy) and disodium methyl arsonate hexahydrate (99.5%) purchased from Chem-Service (USA), respectively. Standards for total As analysis were prepared from 1000 mg L^−1^ As standards supplied by LabKings (Netherlands). Indium and germanium internal standards were prepared from 1000 mg L^−1^ stock solutions purchased from Peak Performance (CPI International, USA).

### Sample and reference materials

A range of brown (Phaeophyta), red (Rhodophyta), and green (Chlorophyta) macroalgae were analysed to ensure the method could be applied to all species of seaweed. During May 2021, samples of *Cystoclonium purpureum* (Rhodophyta) and *Palmaria palmata* (dulse, Rhodophyta) were collected from the intertidal zone of a beach near Kjalarnes, Iceland, and *Porphyra dioicaa* (Rhodophyta) was collected near Grindavík, Iceland. In February 2022, *Fucus vesiculosus*, *Laminaria digitata*, *Saccharina latissima*, and *Ascophyllum nodosum* (at least 10 individuals per species, all Phaeophyta) were collected near Grindavík, Iceland. The samples were transported to the lab in sterile plastic bags, where they were kept refrigerated at 2 °C prior to cleaning. Any epiphytes were manually removed from the seaweed using a stainless-steel knife rinsed with Milli-Q water, and samples were rinsed sparingly with tap water to remove sand. Samples were freeze-dried (Christ, Germany) to constant mass before being milled to a fine powder and stored at room temperature away from sunlight. Brown macroalgae have large thalli with easily distinguishable parts, and so the species of brown seaweed collected were dissected into anatomical parts before drying, e.g. meristem, frond, and reproductive tissues. A detailed description of the sectioning can be found in the Electronic Supplementary Material Fig. S1.

A sample of the tropical seaweed *Asparagopsis taxiformis* (Rhodophyta) was harvested in June 2020, in Azores. A grass silage used for livestock feed (oven-dried, Iceland), freeze-dried mussels (Iceland), and white rice were milled to a fine powder (IKA tube mill, China) before being included in the analysis. Certified reference materials DOLT-5 (dogfish liver), DORM-5 (fish protein), TORT-2 (lobster hepatopancreas), TORT-3 (lobster hepatopancreas), BFLY-1 (blackfly), VORM-1 (worm), and CAME-1 (canola plant) were obtained from the National Research Council of Canada. Hijiki (7405-b, Phaeophyta) which is certified for As(V) was purchased from the National Meteorology Institute of Japan. *Ulva lactuca* (CRM BCR-279, Chlorophyta) was obtained from the Institute of Reference Materials and Measurements in Belgium.

A sample of *A. nodosum* collected in Iceland was used as an in-house reference material for the identification of AsSugars.

### Sample preparation

#### **Total As**

Samples were also analysed for total As. Briefly, 200 mg of material was added to quartz digestion tube with 1 mL HNO_3_ and 1 mL H_2_O_2_ and digested using an ultrawave microwave digestion system. The digestion mixture was then quantitatively transferred to a 50 mL falcon tube and made up to 50 mL with Milli-Q water. A 1 mL of the supernatant from the iAs method was also digested in order to calculate the column recovery.

#### **iAs**

Briefly, 100 mg of freeze-dried ground material was added to quartz digestion tubes with 10 mL of 1% (v/v) HNO_3_ and 3% (v/v) H_2_O_2_ solution. H_2_O_2_ was included in the extraction solution to oxidise As(III) to As(V) so the sum of both inorganic arsenic species could be quantified as As(V), without conversion of organoarsenic species to iAs [17]. The extraction was performed using an ultrawave microwave digestion system (Milestone, Italy), in which the samples were extracted for 40 min at 90 °C before a 15-min cool-down period. The extracts were then transferred to 50 mL falcon tubes and centrifuged (Eppendorf, Switzerland) at 4000 rpm for 15 min. A 1 mL aliquot was transferred to a microcentrifuge tube and centrifuged at 15,000 rpm (Heraeus, USA). The final supernatant was then generally analysed directly using HPLC-ICP-MS but was diluted 1:5 with the extraction solution for CRM hijiki (7405-b) and *L. digitata* frond and sori.

### Analyte quantification

#### **Total As**

Total arsenic measurements were performed using an Agilent SPS4 autosampler and Agilent 7900 ICP-MS with an octopole collision cell in He gas mode. An external calibration in the range 0–200 µg L^−1^ was used for quantification and standards were prepared by serial dilution in 2% HNO_3_. Tuning of the ICP-MS was performed daily, and a 1000 µg L^−1^ indium internal standard was introduced continuously during analysis through a T-piece.

#### iAs

Quantification of iAs was performed using an Agilent Infinity II 1290 HPLC coupled to an Agilent 7900 ICP-MS with octopole collision cell pressurised with He gas (Table 1). The ICP-MS was manually tuned before each use with a 50 µg L^−1^ As solution. Separation of the As species was achieved using a Hamilton PRP-X100 column (250 × 4.1 mm, 10 µm) and corresponding guard column under an isocratic elution with 60 mM ammonium carbonate buffer (3% MeOH). An internal standard containing 50 µg L^−1^ Ge was continuously added post-column during the analysis. The method LOD and LOQ were calculated as 3.3 and 10 times the standard deviation of ten method blanks spiked with 0.1 µg L^−1^ As(V), multiplied by an average dilution factor. Data acquisition and manual integration of peaks were performed using the Agilent Masshunter software. All calculations were performed in Excel.

**Table 1:** The instrument operating parameters for the ICP-MS method used for the quantification of total As and the HPLC-ICP-MS method used for the quantification of iAs

Instrument operating parameters	
ICP-MS settings	Agilent 7900 ICP-MS
RF power	1550 W
RF matching	1.26 V
Plasma gas flow	15.0 L min^-1^
Carrier gas flow	1.07 L min^-1^
Make-up gas flow	0.8 L min^-1^
He gas flow	5.0 L min^-1^
Spray chamber temperature	2 °C
Isotopes monitored	As^75^, In^115^ (internal standard)
HPLC-ICP-MS settings	Agilent 1290 Infinity II HPLC and Agilent 7900 ICP-MS
Isotopes monitored	As^75^, Se^77^, Ge^72^ (internal standard)
Anion exchange column	PRP-X100 (250 × 4.1 mm, 10 µm)
Guard column	PRP-X100 Guard cartridge
Mobile phase	60 mM (NH_4_)_2_CO_3_, 3% MeOH, adjusted to pH 9.0 with ammonia solution
Flow rate	1 mL min^−1^
Injection volume	40 µL

### Method development

The final method (Table 1) used for the extraction and quantification of iAs was optimised using a custom fractional factorial DOE set up in JMP Pro 16 (Table 2) that was used to determine the main effects. The design consisted of 4 factors investigated at 3 levels and 1 factor at two levels: flow rate (0.5, 0.75, and 1 mL min^−1^), buffer concentration (40, 50, and 60 mM ammonium carbonate), HNO_3_ % (0, 1, and 2% v/v) in extraction solution, pH of mobile phase (9, 9.5, and 10), and column length (150 mm or 250 mm PRP-X100, 10 µm). The effects of these 5 factors on both the chromatographic run time and the resolution of the iAs peak to AsSug-SO_4_ were studied. The resolution was calculated using Equation S1 in the Electronic Supplementary Material. Two centre points were also included in the design giving a total of 18 runs that were performed in a randomised order. As the experiments were conducted over two separate days, runs were split into two blocks and block number was included as a variable.

**Table 2:** The parameters for each run of the experimental design and responses. *n* = 1 for all runs with the exception of centre points where *n* = 3

Run	Block	Flow rate (mL min^−1^)	Buffer conc. (mM)	HNO_3_ conc. (v/v %)	pH	Column length (mm)	Runtime (min)	Separation
1	1	0.75	40	0	9	150	9.10	1.08
2	1	0.5	60	0.5	9	150	7.23	1.15
3	2	1	40	1	9	150	4.84	0
4*	1	0.75	50	0.5	9.5	150	4.30	0.41
5	2	0.5	60	1	9.5	150	4.25	0.42
6	2	0.5	40	0	10	150	8.11	1.18
7	1	1	60	0	10	150	3.5	0.49
8	1	0.5	40	1	10	150	6.47	0.39
9	2	1	50	1	10	150	3.54	0
10	2	0.5	50	0	9	250	21.01	3.57
11	1	1	60	0	9	250	11.17	2.09
12	1	0.5	40	1	9	250	14.78	3.20
13	2	1	60	1	9	250	7.43	2.49
14	2	1	40	0	9.5	250	13.05	1.52
15*	1	0.75	50	0.5	9.5	250	11.42	1.93
16	2	0.5	60	0	10	250	14.60	0.80
17	1	1	40	0.5	10	250	7.24	1.93
18	1	0.75	60	1	10	250	9.57	2.18

^*^Centre point

A sample of *A. nodosum* (primary shoot) was chosen to produce the extracts to be used for the DOE due to the high concentration of AsSug-SO_4_. As before, 100 mg of sample material was accurately weighed out into quartz 12 mL tubes before the addition of 10 mL of an extraction solution containing either 0, 1, or 2% (v/v) HNO_3_ and all containing 3% (v/v) H_2_O_2_. The mixtures then underwent extraction as previously described in an ultrawave microwave digestion system and further centrifugation before analysis. The unfiltered extracts were then injected directly.

### Quality control

**Fig. 1:**
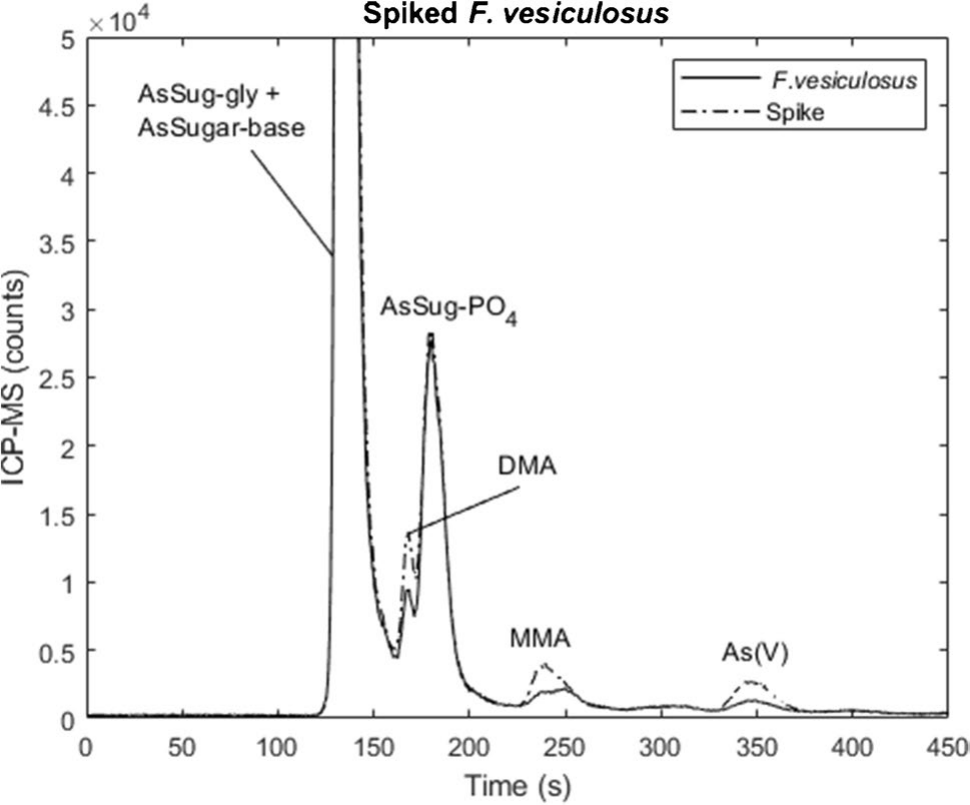
A sample of spiked *F. vesiculosus* extracted using 1% (v/v) HNO_3_ and 3% H_2_O_2_ and analysed with HPLC-ICP-MS. The sample was spiked with 0.5 µg L^−1^ DMA, MMA, and As(V)

Identification of DMA, MMA, and As(V) peaks was carried out by spiking with the respective standards (Fig. 1) and retention time comparison to CRM 7405-b (hijiki) which has a characteristically high As(V) concentration (24.4 mg kg^−1^). Identification of AsSugars was carried out by retention time comparison of an in-house reference material: a sample of *A. nodosum* that had previously undergone LC-MS/MS analysis (Quantiva, Thermo) (Electronic Supplementary Material Fig. S2). Column recoveries were acceptable for all chromatographic runs and calculated to be between 82 and 107%, suggesting all extracted species were sufficiently eluted from the column. Certified reference materials were analysed alongside each batch of samples.

## Results and discussion

### Design of experiment

The resolution of As(V) and the closest eluting peak (which was consistently AsSug-SO_4_ in the sample of *A. nodosum* used) as well as chromatographic runtime were optimised using a custom design in JMP Pro 16. In order to estimate the main effects, the acid concentration in the extraction solution and mobile phase flow rate, pH, and buffer concentration were all studied at 3 levels. Two different column lengths were also evaluated. One sample of *A. nodosum* (primary shoot) was used to produce all extracts used for testing, as this species is known to contain high concentrations of AsSug-SO_4_ which may coelute with As(V) [18]. Extracts were prepared as previously described but with differing HNO_3_ concentrations. The runtime and separation were used as response factors (Table 2), where the runtime was defined as the time the final peak returns to baseline and the separation was defined as the resolution between As(V) and the closest eluting peak to the left.

**Fig. 2:**
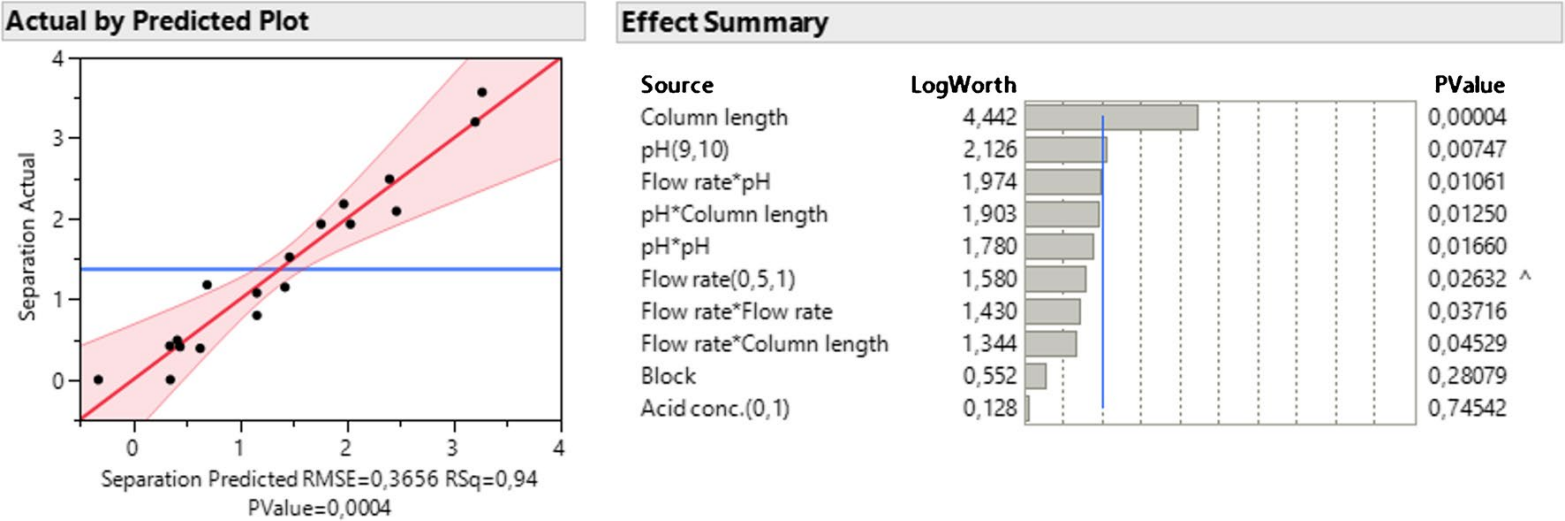
The plot of actual by predicted for the linear regression model for the separation, and summary of effects. The LogWorth values are transformed *p*-values for visual clarity (i.e. LogWorth > 2 is significant at *p* = 0.01 level)

Two separate linear models were generated for each response based on the data in Table 2. It was assumed that all interactions obeyed strong hereditary. The regression model for separation had an *R*^2^ = 0.95 and a low root mean squared error (RMSE) = 0.33, both of which suggest the model adequately describes the observations. The significant factors affecting the separation were found to be the column length and pH of the mobile phase (Fig. 2) where increasing pH was found to have a negative effect on the separation. The term pH*pH being included in the model suggests pH has a parabolic relationship with separation rather than linear. However, as the pKa values of As(V) are 2.19, 6.98, and 11.53 and this experiment is restrained to a pH range of 9–10, linear behaviour can be assumed [31]. This is additionally reflected by the *p* value of the pH*pH term (Fig. 4). Block, acid concentration, and flow rate were not found to be significant factors.

**Fig. 3:**
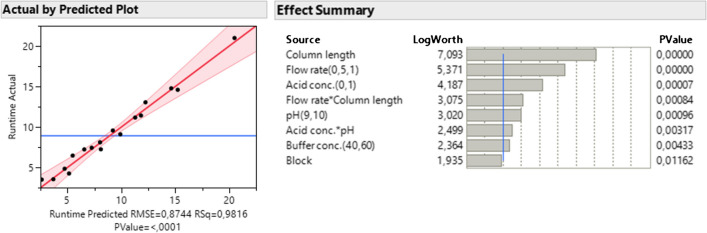
The plot of actual by predicted for the linear regression model for the runtime, and summary of effects. The LogWorth values are transformed *p*-values for visual clarity (i.e. LogWorth >2 is significant at *p* = 0.01 level)

The regression model for runtime had an *R*^2^ = 0.98 and a RMSE = 0.87. This suggests the linear regression is a good fit, but the higher RMSE value means the model may produce less precise predictions than the model for the separation (Fig. 3). Column length, flow rate, pH, buffer concentration, and acid concentration were all found to be significant factors affecting the runtime, where column length had the strongest effect. The block number was not found to be a significant factor.

The separation of As(V) from other analytes was deemed more important than the runtime, and so the significant factors for this response were optimised to maximise the separation, and these results were used as constraints for the optimisation of the runtime. The 250 mm column and pH 9 gave the highest predicted separation (Electronic Supplementary Material Fig. S3). When these values were used as constraints to minimise the runtime, the model predicted the optimised parameters to be a flow rate of 1 mL min^−1^, a buffer concentration of 60 mM, and an acid concentration of 1% (v/v). These parameters were then used for the analysis of all samples. The predicted runtime for these parameters was 6.57 min, and the actual runtime for the seaweed matrices was approximately 6.90 min.

### Method validation

As per Eurachem’s recommendations for method validation, the selectivity, linear range, trueness, and precision of the developed method were tested [32]. The selectivity of the method for iAs measurement was determined by analysis of TORT-3 (lobster hepatopancreas) and hijiki (7405-b) which are confirmed to contain the potential interferent AsSug-SO_4_ [15]. The use of HNO_3_ in the extraction was found to sufficiently remove the interference and allow accurate quantification of iAs, where AsSug-SO_4_ was no longer detectable and the results obtained for iAs in these reference materials were comparable to those previously reported using longer chromatographic methods (Table 3) [15].

**Table 3:** The concentration of DMA, MMA, and iAs (mg kg^−1^) in a range of certified reference materials determined using HPLC-ICP-MS. Concentrations of iAs are compared with those previously reported in literature

Reference material	DMA conc. (mg kg^−1^)	MMA conc. (mg kg^−1^)	iAs conc. (mg kg^−1^)	Total As extracted (mg kg^−1^)	Total As (mg kg^−1^) (certified value)	Extraction efficiency (%)	Column recovery (%)	Reference
DORM-5 (*n* = 5)	0.19 ± 0.02	<LOQ	0.02 ± 0.003 (0.015–0.019)	13 ± 0.31	13.3 ± 0.7	101 ± 2	93 ± 3	[39]
DOLT-5 (*n* = 5)	1.8 ± 0.12	0.10 ± 0.01	0.07 ± 0.007 (0.03–0.04)	32 ± 0.65	34.6 ± 2.4	92 ± 2	97 ± 1	[39]
TORT-2 (*n* = 10)	0.30 ± 0.08	0.08 ± 0.01	0.59 ± 0.04 (0.50)	18 ± 1.4	21.6 ± 1.8	85 ± 7	84 ± 3	[37]
TORT-3 (*n* = 5)	1.1 ± 0.06	0.12 ± 0.02	0.36 ± 0.03 (0.51–0.62)	45 ± 3.4	59.5 ± 3.8	72 ± 6	105 ± 1	[39]
Hijiki (7405-b) (*n* = 5)	0.73 ± 0.10	0.05 ± 0.01	24 ± 2.2 (24.4 ± 0.7)	39 ± 1.4	49.5 ± 1.0	78 ± 3	85 ± 4	[40]
*U. lactuca* (CRM BCR-279)	0.07 ± 0.01	<LOQ	1.4 ± 0.20	2.43 ± 0.46	3.1 ± 0.21	78 ± 14	98 ± 17	-

LOD = 0.006 mg kg^−1^; LOQ = 0.018 mg kg^−1^. Previously reported values for iAs in brackets

The trueness was determined by comparison of results obtained for reference material hijiki (7405-b) with the certified value, where the recovery was found to be acceptable (99 ± 9%). Hijiki is currently the only marine reference material with a certified value for iAs; therefore, spiking recovery experiments were performed with a sample of *F. vesiculosus* known to contain high concentrations of AsSug-SO_4_ relative to the iAs concentration [18]. Spiking with 1, 5, and 10 µg L^−1^ yielded acceptable recoveries of 112, 92, and 101% respectively. Additionally, to rule out any matrix effects, an internal calibration was also performed for the same sample in triplicate, where samples were spiked at 3 levels (0.5, 5, and 10 µg L^−1^) using an As(V) standard. No significant difference was found between the results obtained using an internal calibration vs. an external calibration (*p* = 0.46), where concentrations were calculated as 0.054 ± 0.009 mg kg^−1^ and 0.055 ± 0.004 mg kg^−1^, respectively.

Precision is defined as the repeatability (within batch or run) and the reproducibility (between laboratories). As this study is a single-laboratory validation, the reproducibility was not tested; however, samples showed good repeatability between replicates where RSD between replicates of reference materials ranged from 7 to 15%. The LOD achieved was comparable to others reported [14, 25, 33] and is considered acceptable for the determination of iAs in rice products to comply with European legislation, whereby levels must not exceed 0.04 mg kg^−1^ for white rice [34]. Additionally, calibration within the working range of the method was shown to be linear (Electronic Supplementary Material Fig. S4).

### Reference materials

There is a clear lack of consensus in the literature over the value of iAs in marine reference materials solutions—with multiple sources reporting values with order of magnitude difference for the same material. For the reference material TORT-2, iAs values as low as 0.09 mg kg^−1^ and as high as 1.23 mg kg^−1^ have been reported when using HPLC-ICP-MS [35–37]. This study quantified the iAs concentration in TORT-2 as 0.586 mg kg^−1^ which is well within this range of values (Table 3) and similar to values obtained using a microwave-assisted extraction, an acidic solvent and subsequent quantification with LC-ICP-MS (0.50-0.78 mg kg^−1^) [37]. The results for TORT-3 were lower than those reported using an extraction solution containing methanol (0.631 mg kg^−1^) [15] and also those using HNO_3_ in the extractant (0.43 mg kg^−1^) [33], and were more similar to results from extraction procedures using water as the solvent (0.341 mg kg^−1^) [38]. A similar trend is observed for DOLT-5, where extractions using aqueous methanol report greater iAs values (0.22 mg kg^−1^) in comparison with this study (0.068 mg kg^−1^). Previous research has reported DOLT-5 iAs concentrations in the range of 0.029–0.076 when using low concentrations of HNO_3_ and/or H_2_O_2_ [33, 39]. Concentrations of iAs in DORM-5 and hijiki were in good agreement with certified or previously reported values.

MMA concentrations were generally lower than reported values for all reference materials, possibly due to partial separation from an unknown compound (UK) (Fig. 1). DMA values were slightly lower than reported previously, with the exception of hijiki which was consistently higher than the literature values, 0.73 ± 0.10 mg kg^−1^ compared to 0.24 ± 0.01 mg kg^−1^ [39, 40]. This increase in DMA is unlikely the result of AsSugars degradation as the breakdown is acid-induced rather than microbial. Thus, the reaction would be expected to proceed via the base AsSugar (Fig. 2) rather than the DMA and other intermediates observed during microbial degradation [16, 41]. Higher marine organisms may also contain AsSugars and a similar trend was not observed in these matrices. The lower concentrations of DMA reported in the other marine reference materials, e.g. DOLT-5, may potentially indicate that a compound unstable under acidic conditions is coeluting with DMA; for example, dimethylarsinoyl acetate (DMAA) is often present in higher marine organisms but not algae, and has been shown to have a similar retention time to DMA [15, 38].

All reference materials showed good extraction efficiencies (72–101%); however, the value for hijiki is higher than previously reported with milder aqueous extractions (42–53%) [40, 42]. The use of acid in the extraction solution may have potentially caused the degradation of arsenic-containing lipids to water-soluble species, as Hijiki *spp.* have been shown to contain significant concentrations of lipid-soluble As; however, this requires further investigation [43]. Similar extraction efficiencies are observed for marine animal matrices regardless of the use of acid in the extraction, as the main arsenic-containing lipid species are fatty acids and hydrocarbons [44]. High extraction efficiencies have been reported for the *U. lactuca* CRM when HNO_3_ was used in the extraction solvent [45]. The same study reported significantly lower values for DMA (0.04 mg kg^−1^); however, a shorter extraction time was used, and a value of 0.08 mg kg^−1^ for DMA was reported when a methanol/water extraction solution was used. Additionally, the authors report a value of 1.2 mg kg^−1^ for As(V) (this study: 1.36 mg kg^−1^) and did not detect AsSug-SO_3_ or AsSug-SO_4_ [45].

TORT-2 showed large differences in extraction efficiency dependent on the sample weight used, and so the effects of two sample-to-solvent ratios on analyte concentration and total As were investigated (Table 4). Significantly higher concentrations of total As were extracted when less sample material was used, as well as significantly higher levels of iAs. The same trend was not observed for other analytes extracted from TORT-2 suggesting that either the iAs binding differs from the other arsenic compounds, or that the As(V) is in a form with a low solubility coefficient and is thus not extracted quantitatively when high sample weights and low solvent volumes are used.

**Table 4:** The iAs and total As concentrations in TORT-2 from two different sample-to-solvent ratios

Sample to solvent ratio (mg:mL)	*n*	iAs (mg kg)*	Tot As extracted (mg/kg)*	Extraction efficiency (%)*
50:10	5	0.63 ± 0.04	19.68 ± 0.62	91 ± 3
100:10	5	0.52 ± 0.03	17.25 ± 0.83	80 ± 4

^*^Significant difference between ratios (*p* < 0.05)

### Seaweed samples

The developed method worked well for all species of seaweed tested, with no interferences observed from the high concentrations of AsSug-SO_4_ that can be present in some species of brown seaweeds [46]. The *L. digitata* reproductive tissues (sori) contained the highest levels of iAs (54 mg kg^−1^), followed by the *L. digitata* meristem and *A. taxiformis* (Table 5). The iAs concentration of all seaweed samples collected in Iceland was found to be below the maximum levels set by France for seaweed-based condiments (3 mg kg^−1^) with the exception of the *L. digitata* sori, which exceeded this by many multiples. Levels of MMA in the seaweed samples varied between <LOQ and 1.5 mg kg^−1^, where the Laminariales typically had the highest concentrations. The concentrations of DMA ranged from 0.16 to 1.1 mg kg^−1^. The extraction efficiency was particularly low for *C. purpureum* (49%) and several brown seaweed samples (Table 5). Low extraction efficiencies have also been reported for species of red seaweed collected off the coast of China, where as little as 15.4% of the total arsenic from *Eucheuma denticulatum* was extracted using water [47]. It may be that the As is not in water-soluble form, i.e. arsenic-containing lipids or As(III) strongly bound to sulphur residues in proteins, so will not be extracted using aqueous extraction solvents. Brown seaweed species collected in winter months have previously been reported to contain lower amounts of water-soluble As than those found in summer [48].

**Table 5:** The concentration of iAs (mg kg^−1^) in seaweed samples

Seaweed (*n* = 3)	DMA conc. (mg kg^−1^)	MMA conc. (mg kg^−1^)	iAs conc. (mg kg^−1^)	Total As extracted (mg kg^−1^)	Total As (mg kg^−1^)	Extraction efficiency (%)	Column recovery (%)
*P. dioica*	0.16 ± 0.02	<LOQ	0.05 ± 0.00	24 ± 0.62	22 ± 0.93	111 ± 3	99 ± 3
*A. taxiformis*	0.73 ± 0.07	0.14 ± 0.01	2.4 ± 0.03	13 ± 1.4	14 ± 0.17	92 ± 10	100 ± 3
*P. palmata (Dulse)*	0.23 ± 0.03	<LOQ	0.36 ± 0.01	7.9 ± 2.7	8.3 ± 0.21	95 ± 18	92 ± 12
*C. purpureum*	0.18 ± 0.02	<LOQ	0.45 ± 0.01	2.8 ± 0.40	5.6 ± 0.16	49 ± 7	105 ± 13
*F. vesiculosus* (apice)	1.4 ± 0.14	0.38 ± 0.04	0.35 ± 0.06	72 ± 10	78 ± 0.74	92 ± 13	107 ± 6
*F. vesiculosus* (blade)	0.31 ± 0.04	0.13 ± 0.01	0.08 ± 0.01	21 ± 3.7	32 ± 4.5	66 ± 12	105 ± 9
*A. nodosum* (primary shoot)	0.52 ± 0.04	0.03 ± 0.02	0.05 ± 0.01	19 ± 2.6	33 ± 1.1	59 ± 8	98 ± 2
*A. nodosum* (reproductive receptacle)	0.77 ± 0.06	0.14 ± 0.02	0.10 ± 0.02	48 ± 0.61	113 ± 23	43 ± 1	105 ± 2
*L. digitata* (meristem)	0.73 ± 0.06	1.3 ± 0.40	2.6 ± 0.01	97 ± 1.5	130 ± 7.5	74 ± 1	94 ± 3
*L. digitata* (sori)	0.31 ± 0.04	0.35 ± 0.06	54 ± 1.3	85 ± 3.9	210 ± 25	40 ± 2	107 ± 2
*S. latissima* (stipe)	0.63 ± 0.03	0.54 ± 0.04	0.12 ± 0.01	49 ± 1.1	76 ± 3.4	65 ± 1	100 ± 3
*S. latissima* (frond)	0.71 ± 0.05	1.5 ± 0.43	0.05 ± 0.01	142 ± 9.3	214 ± 27	66 ± 4	105 ± 6

^*^Literature value [45], LOD = 0.006 mg kg^−1^, LOQ = 0.018 mg kg^−1^

As previously discussed, the concentrations of DMA detected in seaweed matrices increased with the use of acid in the extraction solution. This trend was investigated further by performing two separate extraction and analysis methods to quantify arsenic species in one sample *of A. nodosum* (Table 6). The use of an acidic extraction solution results in higher concentrations of DMA than the use of a water/H_2_O_2_ solution, but the increase does not appear to arise from the degradation of AsSugars. AsSug-SO_4_ appears to degrade to the base sugar resulting in a quantitative increase in the peak associated with AsSug-gly, and AsSug-SO_3_ now appears to coelute with AsSug-PO_4_ [16]. Thus, it is likely that the increase in DMA is the result of an additional compound being extracted and degraded—such as a lipid species unique to seaweed. Although further analysis with mass spectrometry would be needed to confirm the exact products created during extraction, previous studies have reported DMA as a product of lipid degradation in seaweed [49]. It could also be argued that the acidic extraction provides a closer estimation of the DMA concentration that individuals would be exposed to after the consumption of seaweed, as similar concentrations of HNO_3_ have been previously used to simulate gastric juice [16].

**Table 6:** As speciation of *A. nodosum* (primary shoot) sample collected from the Grindavík location in May 2021 using two separate extraction methods and analyses

	Method 1 (*n* = 2)	Method 2 (*n* =3)
Extraction solvent	3% (v/v) H_2_O_2_	1% HNO_3_ (v/), 3% (v/v) H_2_O_2_
Extraction method	Mechanical shaking RT Overnight	Microwave-assisted 90 °C 40 min
HPLC method	Gradient Ammonium carbonate 0.5 mM, 50 mM (3% MeOH, pH 9.2)	Isocratic Ammonium carbonate 60 mM (3% MeOH, pH 9)
Total As extracted (mg kg^−1^)	15.31	18.13
AsSug-gly (mg kg^−1^)	3.00	12.84*
DMA (mg kg^−1^)	0.08	0.41
AsSug-PO_4_ (mg kg^−1^)	1.17	4.07
AsSug-SO_3_ (mg kg^−1^)	1.81	ND
AsSug-SO_4_ (mg kg^−1^)	9.44	0.22
iAs (mg kg^−1^)	0.06	0.05

^*^Sum AsSug-gly and base sugar (no side chain). The base sugar has been demonstrated to elute at same time as AsSug-gly [16, 45]. *ND* not detected

### Application to other matrices

**Fig. 4:**
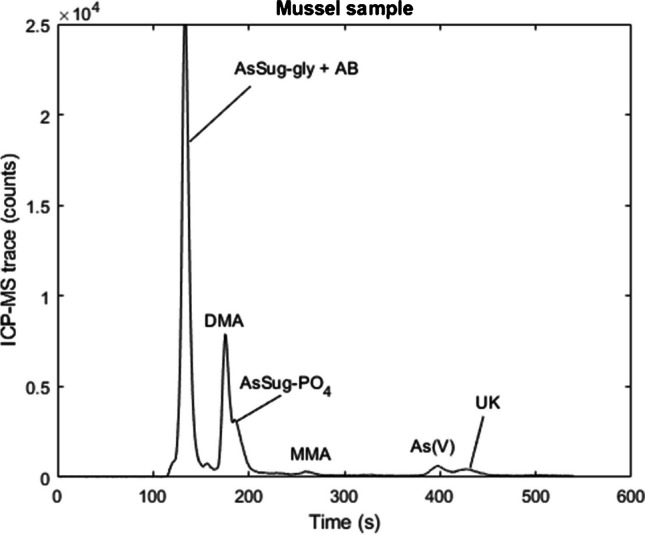
A chromatograph of mussel sample extracted with 1% (v/v) HNO_3_ and 3% H_2_O_2_ solution and analysed with HPLC-ICP-MS

To test ruggedness, the developed method was applied to other marine and terrestrial matrices. All terrestrial samples and the mussel sample contained reproducibly low levels of iAs, ranging from 0.018 to 0.15 mg kg^−1^ (Table 7). In the grass-based feed and CAME-1, As(V) was the only analyte detected, and in VORM-1, small cationic peaks were additionally detected in the void volume. Although reference materials VORM-1, BFLY-1, and CAME-1 were only recently introduced, the results are in good agreement with values published from an inter-laboratory study, with the exception of BFLY-1 which is higher than previously reported [39]. The concentration of arsenicals detected in the rice sample is typical of this matrix [50–52], and the mussel sample contained relatively high levels of DMA and low levels of MMA along with several AsSugars (Electronic Supplementary Material Table S1). An unidentified late-eluting compound was also detected in the mussel sample (Fig. 4) similar to an unknown reported in blue mussels from Norway [15], where the authors used a methanol/water extractant. The extraction efficiencies were high with the exception of CAME-1, where less than half of the total arsenic was extracted in the form of As(V). The recalcitrant arsenic is likely to be in the form of As(III) bound to sulphur-containing proteins rather than lipid-soluble species, as CAME-1 is comprised of the high protein residue left over after the extraction of canola oil from *Brassic*a *napus* [39, 53].

**Table 7:** The iAs concentration (mg kg^−1^) of other matrices quantified by the developed method

Sample material (*n* = 3)	DMA conc. (mg kg^−1^)	MMA conc. (mg kg^−1^)	iAs conc. (mg kg^−1^)	Total As extracted (mg kg^−1^)	Total As (mg kg^−1^)	Extraction efficiency (%)	Column recovery (%)
Grass silage (feed)	ND	ND	0.03 ± 0.00	0.03 ± 0.00	0.04 ± 0.0	72 ± 7	107 ± 12
Rice	0.10 ±	<LOQ	0.15 ± 0.01	0.25 ± 0.03	0.29 ± 0.01	89 ± 10	101 ±10
Mussel	1.2 ± 0.04	0.05 ± 0.00	0.15 ± 0.00	7.08 ± 0.04	13	55 ± 0.3	91 ± 2
Worm (VORM-1)	ND	ND	0.09 ± 0.00 (0.11^A^)	0.10 ± 0.01	0.13^A^ ± 0.00	75 ± 7	100 ± 3
Blackfly (BFLY-1)	<LOQ	<LOQ	0.08 ± 0.01 (0.05^A^)	0.09 ± 0.01	0.10^A^± 0.00	88 ± 12	90 ± 5
Canola plant (CAME-1)	ND	ND	0.02 ± 0.00 (0.01^A^)	0.02 ± 0.00	0.04^A^	64 ± 4	94 ± 9

^A^Literature value [39]; LOD = 0.006 mg kg^−1^; LOQ = 0.018 mg kg^−1^. *ND* not detected

## Conclusion

Routine measurement of the iAs levels in seaweed is likely to become crucial in the future as European legislation is expanded to include these matrices—highlighting the need for accessible methods for routine analysis. The method developed using DOE is suitable for the high-throughput analysis of iAs in a range of matrices (i.e. seaweed, rice, insect and feed), and can provide adequate separation of compounds from As(V) in a similar chromatographic time frame to that of HG-ICP-MS [33]. The use of HNO_3_ and H_2_O_2_ allows for a “partial speciation” approach where iAs is quantified as the sum of both inorganic species oxidised to As(V) and interferences from high levels of AsSugars are removed by degradation to an unretained AsSugar species. The method produced accurate and repeatable results, where the recovery of iAs in hijiki was 99 ± 9% when compared with the certified value and spiking experiments with *F. vesiculosus* yielded acceptable recoveries (92–112%). The applicability of this method to a diverse range of matrices allows for a “one-fits-all” extraction and analysis that is cost-effective, fast, and robust and requires minimal sample preparation. However, this method is limited in its determination of DMA in seaweed matrices, and care should be taken when using HNO_3_ in the extraction solution if the aim is to quantify this analyte, as this may provide an overestimation by degrading unknown As species. However, the concentrations of DMA quantified using this method may provide a better estimation with regard to exposure after ingestion and subsequent digestion of seaweed. Further research should investigate the application of this method to other food matrices which are currently regulated (i.e. fruit juice concentrates and infant foods) [34].

### Supplementary Information

Below is the link to the electronic supplementary material.

Supplementary file1 (DOCX 1.59 MB)
