# Supplementary material for: Inorganic arsenic in seaweed: a fast HPLC-ICP-MS method without coelution of arsenosugars
Source: Anal Bioanal Chem. 2024 Mar 23;416(12):3033–44. doi: 10.1007/s00216-024-05250-8 (PMC11045606; doi:10.1007/s00216-024-05250-8)
Supplement: Supplementary file 1 — Supplementary file1 (DOCX 1.59 MB) [file 216_2024_5250_MOESM1_ESM.docx]

**Electronic Supplementary Material**

**Inorganic arsenic in seaweed: a fast HPLC-ICP-MS method without coelution of arsenosugars**

Rebecca Sim­ ^A B^, Marta Weyer ^C*^ and Ásta H. Pétursdóttir ^A*^

Author Affiliations:

^A^ Public Health and Food Safety, Matís, Vínlandsleið 12, 113, Reykjavík, Iceland

^B^Faculty of Physical Sciences, Dunhagi 3, 107, University of Iceland, Reykjavík, Iceland

^C^Department of Chemistry, University of Aberdeen, Meston Walk, Aberdeen, Scotland

*Correspondence to: [asta.h.petursdottir@matis.is](mailto:asta.h.petursdottir@matis.is); marta.weyer44@gmail.com

| **Table of contents** |
| --- |
| Fig S1…………………………………………………………………………………………………Page 2 |
| Fig S2…………………………………………………………………………………………………Page 4 |
| Fig S3……………………………………………………………………………………………….…Page 4 |
| Table S1…………………………………………………………………………………………….…Page 5 |
| Equation S1………………………………………………………………………………………….…Page 6 |
| Fig S4…………………………………………………………………………………………………Page 6 |

| ***Ascophyllum nodosum*** |
| --- |
| 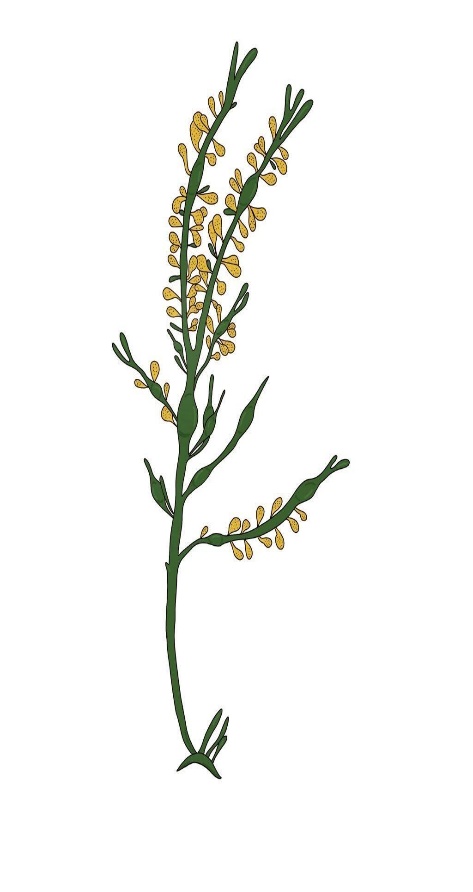 Reproductive receptacles  Holdfast and stipe  Secondary shoots  Primary shoot |
| ***Fucus vesiculosus*** |
| 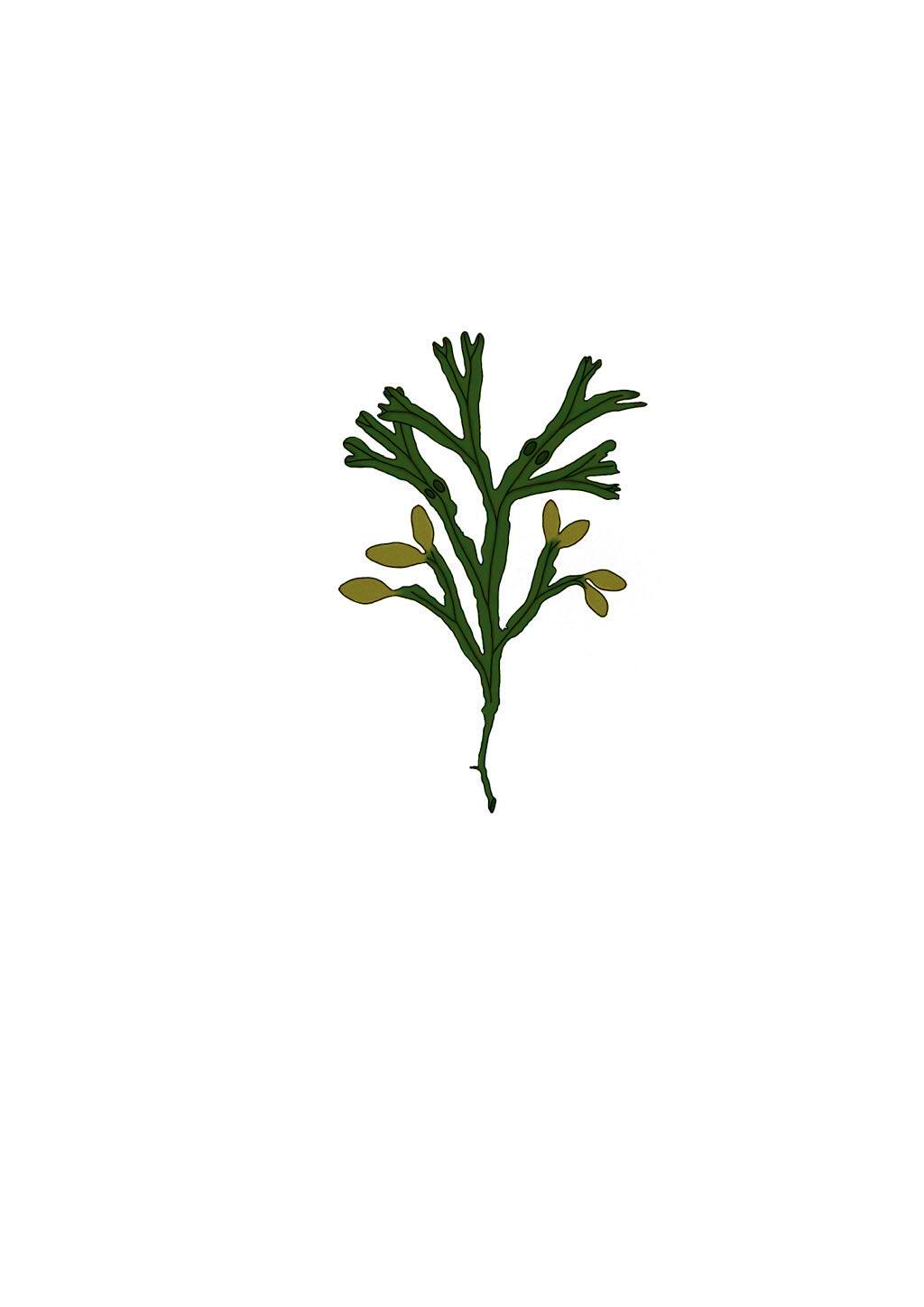 Apices  Bladders  Reproductive receptacles  Blade  Holdfast and stipe |
| ***Laminaria digitata*** |
| 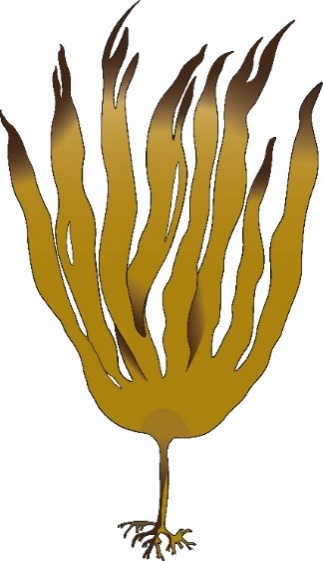 Decaying frond  Old frond  Meristem  Holdfast/stipe  Young frond  Sori (reproductive tissue) |
| ***Saccharina latissima*** |
| 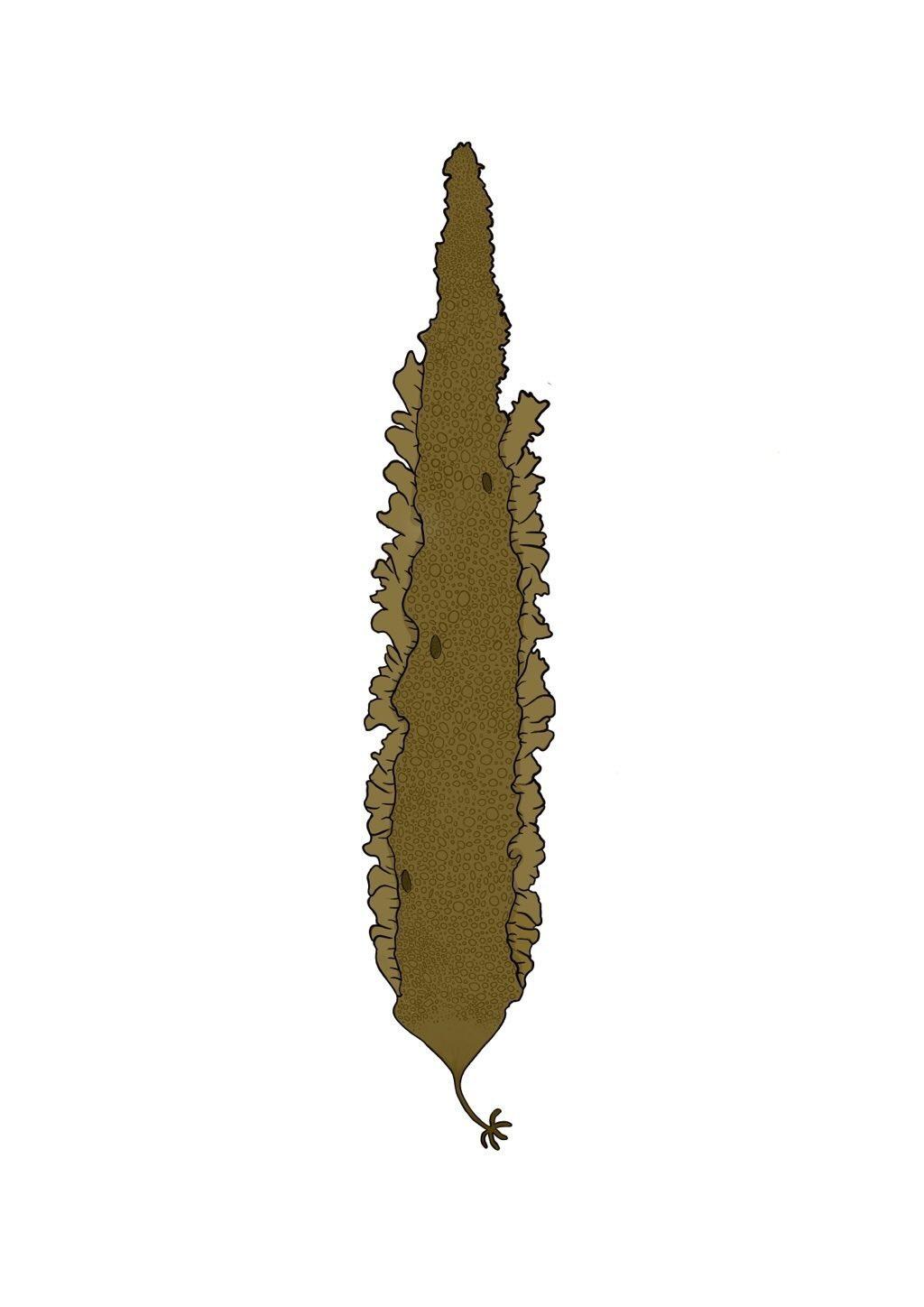 Holdfast and stipe  Sori (reproductive tissue)  Meristem  Young frond  Old frond |

Fig S1. The division of brown seaweed thalli into anatomical sections.

Fig S2. The identification of arsenosugars in a sample of *Ascophyllum nodosum* used as an in-house reference material by LC-MS/MS (Quantiva, Thermo).


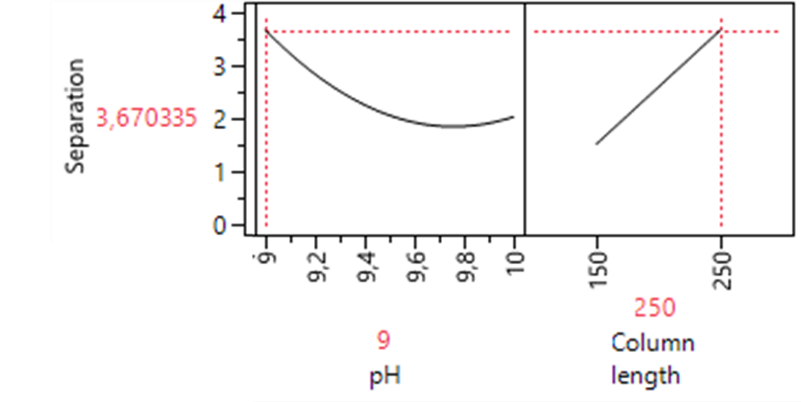

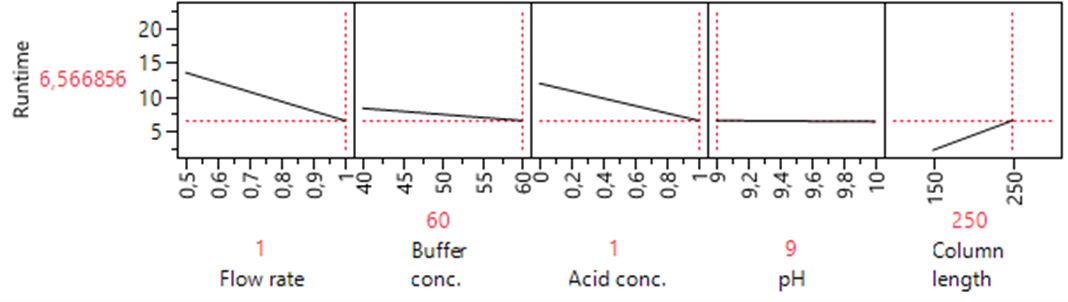


Fig S3. The prediction plots for the significant factors affecting the separation and runtime.

Table S1. All results from the seaweed and mussel samples analysed.

| Sample | AsSug-gly  (mg kg^-1^) | DMA  (mg kg^-1^) | AsSug-PO4  (mg kg^-1^) | MMA  (mg kg^-1^) | iAs  (mg kg^-1^) | Sum of unknown (mg kg^-1^) | Total As extracted (mg kg^-1^) | Column recovery (%) |
| --- | --- | --- | --- | --- | --- | --- | --- | --- |
| Sea lettuce (CRM BCR-279 | 0.822 ± 0.161 | 0.071 ± 0.006 | ND | <LOQ | 1.359 ± 0.198 | - | 2.425 ± 0.455 | 98 ± 17 |
| *P. dioica* | 21.885 ± 0.513 | 0.163 ± 0.016 | 1.295 ± 0.150 | <LOQ | 0.050 ± 0.003 | 0.172 ± 0.014 | 23.938 ± 0.621 | 99 ± 3 |
| *A. taxiformis* | 7.542 ± 0.581 | 0.731 ± 0.0723 | 2.00 ± 0.355 | 0.141 ± 0.008 | 2.382 ± 0.027 | 0.173 ± 0.012 | 13.003 ± 1.419 | 100 ± 3 |
| *P. palmata (Dulse)* | 5.162 ± 0.420 | 0.234 ± 0.033 | 1.298 ± 0.191 | <LOQ | 0.361 ± 0.013 | 0.030 ± 0.003 | 7.905 ± 2.725 | 92 ± 12 |
| *C. purpureum* | 1.426 ±0.064 | 0.184 ± 0.019 | 0.748 ± 0.029 | <LOQ | 0.450 ± 0.013 | 0.043 ± 0.002 | 2.778 ± 0.399 | 105 ± 13 |
| *F. vesiculosus* (apice) | 63.167 ± 6.129 | 1.376 ± 0.140 | 11.112 ± 0.929 | 0.376 ± 0.035 | 0.352± 0.056 | 0.450 ± 0.114 | 72.085± 10.258 | 107 ± 6 |
| *F. vesiculosus* (blade) | 17.068 ± 1.674 | 0.307 ±0.041 | 3.878 ± 0.568 | 0.133 ± 0.014 | 0.082 ± 0.013 | 0.137 ± 0.002 | 20.844 ± 3.685 | 105 ± 9 |
| *F. vesiculosus*  (reproductive tip) | 48.913 ± 1.102 | 0.453 ± 0.044 | 7.375 ± 0.533 | 1.166 ± 0.393 | 0.197 ± 0.020 | 0.514 ± 0.068 | 50.484 ± 7.776 | 120 ± 17 |
| *A. nodosum* (primary shoot) | 12.786 ± 1.030 | 0.517 ± 0.038 | 4.895 ± 0.994 | 0.030 ± 0.015 | 0.059 ± 0.013 | 0.274 ± 0.049 | 19.357 ± 2.558 | 98 ± 2 |
| *A. nodosum* (reproductive receptacle) | 40.393 ± 0.479 | 0.766 ± 0.058 | 7.690 ± 0.111 | 0.139 ± 0.023 | 0.101 ± 0.017 | 1.448 ± 0.168 | 48.264 ± 0.613 | 105 ± 2 |
| *A. nodosum*  (secondary shoot) | 17.829 ± 1.998 | 0.407 ± 0.026 | 4.760 ± 0.611 | 0.036 ± 0.014 | 0.060 ± 0.003 | 0.407 ± 0.090 | 22.053 ± 1.403 | 101 ± 4 |
| *L. digitata* (meristem) | 81.880 ± 1.890 | 0.730 ± 0.064 | 3.314 ± 0.353 | 1.259 ± 0.402 | 2.599 ± 0.012 | 1.039 ± 0.063 | 96.528 ± 1.453 | 94 ± 3 |
| *L. digitata* (sori) | 25.177 ± 0.545 | 0.311 ± 0.035 | 10.829 ± 0.415 | 0.352 ±0.058 | 54.203 ± 1.343 | 0.260 ± 0.021 | 84.889 ± 3.890 | 107 ± 2 |
| *L. digitata*  *(old frond)* | 42.876 ± 0.484 | 0.635 ± 0.015 | 5.015 ± 0.677 | 0.600 ± 0.090 | 77.917 ± 3.275 | 0.329 ± 0.005 | 134.439 ± 7.157 | 95 ± 7 |
| *L. digitata*  (decaying distal frond) | 27.500 ± 0.984 | 0.510 ± 0.067 | 5.651 ± 1.347 | 0.409 ± 0.034 | 80.044 ± 1.574 | 0.252 ± 0.025 | 108.031 ± 6.423 | 106 ± 4 |
| *S. latissima* (stipe) | 41.621 ± 0.717 | 0.633 ± 0.025 | 6.333± 0.223 | 0.542± 0.036 | 0.116± 0.006 | 0.336 ± 0.019 | 49.450 ± 1.082 | 100 ± 3 |
| *S. latissima* (old frond) | 137.006 ± 2.248 | 0.713 ± 0.045 | 8.044 ± 0270 | 1.501 ± 0.433 | 0.0463 ± 0.006 | 0.863 ± 0.038 | 141.736 ± 9.298 | 105 ± 6 |
| *S. latissima*  (young frond) | 118.147 ± 1.152 | 0.448 ± 0.009 | 7.037 ± 0.523 | 0.861 ± 0.032 | 0.035 ± 0.002 | 0.849 ± 0.089 | 112.030 ± 11.7523 | 115 ± 11 |
| *S. latissima*  (meristem) | 59.417 ± 0.123 | 0.253 ± 0.012 | 2.932 ± 0.026 | 1.176 ± 0.095 | 0.027 ± 0.003 | 0.236 ± 0.037 | 63.430 ± 3.437 | 101 ± 5 |
| *S. latissima* (sori) | 139.006 ± 2.428 | 0.467 ± 0.046 | 6.053 ± 0.277 | 2.071 ± 0.196 | 0.053 ± 0.002 | 1.430 ± 0.160 | 138.037 ± 13.769 | 109 ± 13 |
| Mussels | *4.443 ± 0.041 | 1.18 ± 0.04 | 0.481 ± 0.020 | 0.05 ± 0.002 | 0.150 ± 0.002 | 0.142 ± 0.020 | 7.083 ± 0.039 | 91 ± 2 |

*Sum of AsSug-gly and arsenobetaine (AB). AB is not known to be produced by seaweeds.

$$R=\frac{(RT2-RT1)}{0.5*(W2+W1)} eq (1)$$

Equation 1. Where R is the chromatographic resolution of two peaks. RT is the retention time of a peak and W is the width of a peak.

Fig S4. The range of linearity (top) and residual plot for the working range (bottom) of the developed method. The range of linearity is between 0-200 ppb, and after this the response of the instrument appears to decrease (lower slope). The working range is 0-100 ppb, and the residuals (y observed – calculated y value) show a random distribution around 0 suggesting the working range is linear.
